# Supplementary material for: The microRNA Pathway of Macroalgae: Its Similarities and Differences to the Plant and Animal microRNA Pathways
Source: Genes (Basel). 2025 Apr 9;16(4):442. doi: 10.3390/genes16040442 (PMC12026948; doi:10.3390/genes16040442)
Supplement: Supplementary file 1 [file genes-16-00442-s001.zip › genes-3560056-supplementary.pdf]

Table S1: Amino acid sequences of candidate machinery proteins of the *Asparagopsis taxiformis* miRNA pathway.

Asparagopsis taxiformis DCL3 (ATA00637) (1179 amino acid residues)
MTATPVKAKASAKSEQPCLKAFAALEATLDATVVTVSPESQSEVEVMVPKPEEFVITYQSKSTVEDTELEPRD<br>EFEASESAAEVIALMAASEANGESAKLPSHVQEGVLHFAEINLLNCTELSARSIVGAQSAESHVRMSQSKQNS<br>IIKDFRDGNFGILVATNVVEEGLDVPACRLVVAFDSVMSPTAYVQGRGRARKRGARYIVFLEEDSLQHYLALY<br>YAREGAKLMKDVANGEGCPEAERELCRKKLFEESVMTERKLYSRTTRARVGATEAVNLLHRYCFMKSAELGDI<br>PFQKPCYLPKESPEGYTAIVTMHPRIPIDSGICSVPQCNPAIAKRYAALDAYTKLYQIGEVDEYLLPKRPARS<br>KRVLHVIKPRSDLGVRIQVQRREQKAHSEMQPKASKKDKRLRVCRIAHPGPLQVKLADVPSENLEPSLNPING<br>TKNDHGGLEKFESRRITIAEKASCSSLFMYSIRLDHDVKSSSWYRPEDDGTFGLLLREKVLTEDLSAIRCPFG<br>QDLLTLSYEGSIPWTESYQKTGYKYCRYMQLCLRGRCPGSIAAQEIEQREYGSSISTGFLLLPLMKIYDSGSP<br>IAYDIDWGSIQQLLSLDWRSGNRAIAQELQRALVCSDHENCERVYLSSELDEQIKAKSSSRGYLNTAYRSFKQ<br>YFAEKHSTPLEDENQSMLATCNPLDMFSRTSVSTFMLPPETCRLIPLSPMACYIASVLPTWQTFLALRNCWRR<br>NRIESNPIEFLAFARALQPNVNNVSKGCVDLSYERLEFLGDAVLKVINSMVSFVQTPEESEGRLSDSRDREVC<br>NKNLADYAIESKIQDCVAFSGVSQKAKAWPWFWGAQQNKCVEISEKVLADCVESIIGVQYLHGGIELATEFLD<br>LLTLTKGACEVLGIVKGGKKRENGRINVHLPVMGTGDKRYESAFVKEVEEIIGYEFVNKNHLVVALTHGSYLN<br>GRITSYQRYEYLGDAIVGFLLLSHFFDKYPKLSPGELTSLRGPALSNDLFARVVVSWGIHKRFWFECPPLVTE<br>IRKFEELVANEDDEEDVCKTITVPKVLGDLLESIIGAVVVDKGMRFDGVKAIVLRLMESELERFANPDKFKHN<br>PVSEMVRMVQKLYNTHPTYHYLDGARDVVKICSIEVDKREIGRGTGPTRRVAKHKAAIEGLKVLGEEEGNGDG<br>EREDLPCETSK
Asparagopsis taxiformis SE1 (ATA06899) (1054 amino acid residues)
MPIHMQRPQNPQPNLPVIQPQYPQYQQSSSQPGPNPTYTRQRDWDPSREDYERDPKRMRPFPQNSSEPPYPYN<br>QPRPDIRVPQQISHHTPAPQSASYNQITPHTSHVPPNAPSIHPPSQHPPYPPRQVPFAQQPVLSSHQSQPDYA<br>YPPKPAAPYPPQQQPNVPDNYDRLPAPPRKDFHDPAQQPIPPVADPEHKPEHKAEPKDEQDKPMSFKDFLAMQ<br>PDSISPQAAQEAFDEYVRDFTRRKPNKFFDLHKDEEWFRERYDPEYVSRRISRIEVEVNERAKVYKKLWDKGG<br>SKVCAPKLTVGPEPAKQHSSQPGAQSGKSSNQSSPEQGEHSDTDDQGTAAMKDEAMMLIHDALKDEDKDTGET<br>IPGRKAEQEPTSGDNKTDLDKSNKAKDELKMDVPDGAKTEAKAEAADMEEKAPKPEVEMDTEEVPKEEKQLLD<br>IESTVTDIQLAKDESDNSRPEEAKEEAQDKKNPEKDEPQVKKAEAIVASGGSEDTDGDAAGLVLPLRREHQKD<br>TIFMRGIPLNLARDALTEVLKHGPEGKHSFNLRRLKLGDINPVRSLQRFGWAVYDSEETAGRALDIVRGVVVR<br>SPPEDKKEAESSKGDDDESKSPVEDDPSWIYEIDCMLNLERRKKFTMGRVLPAPFGTSDRMEHDVEQSVKMMR<br>SLDSLRKIDEDLNPLTDEFLNGLQSDGDRLDHIVTYLREVHYFCYYSGNEFLEDPTSMPPQELRPSGERTRSS<br>ICEADSRLLRRVDERAKWVLERDYDRPRSNSDNGEAQKDEAIQQWLDSNTKNEGQGRYRCGLPPHKLFKGPEF<br>VHKHLRTKHGDVMKEVIDKALMETYRSNFENDASKTEVIAIFDEGMAGNLDDSKGTGAKHLGTAGAGSQSHGY<br>SGIGEAYNQGRTNMPVGMYNAASPFMGMGIQPFPMMMMSTPGFPTGYSTPGYGNNMSFNGRGMSGNGMGGVGM<br>GRMGGGGMMTGGAKGGDVSNMAMQRRSNVRGRDGGHREGGFREYRESGYRDSGGHRGRRGHPRRGGGEAHRRP<br>LDPRASGPRRSYNDLDAPANGPSFDLVRYEDV
Asparagopsis taxiformis XPO5 (ATA01138) (1107 amino acid residues)
MDRLADPNLPDDQYVKLLEQPVAILYTSPNAAERFAAQSLLTTIQELPNAWVRVDKVLDYPASSSNAKFFALQ<br>ILEKLIRYRWKTLQRSTCESIRNYVVNKVIKLSETDESLNRERVFLRKLNLILVQIVKQEWPAKWESFMTEIV<br>GASRSSVSLCENNMKILRLLSEEVFEFSNGQMTQDKIVELKKQFNDDFSRVFQLCQYVFTSTADLQKTRPALL<br>VATLNTLEKFLSWIPLGYIFETQLIETLVGFMSVPALRHSALPCLVEIASLSVTNTYDDRFRILFVAFVKQLV<br>NVLPRDTDIAAAYEDSNDEVQGFVMDLALFFTGFFRAHISLLDVDGPPDIQEALRVAHEYLVKISRVSEVEVF<br>KTCLEWWFRLASDLYDNECHIPPGADRNRPLLLGNARDHPPAVIGMAGVSGPAAAITVSPKRSFYAPILSEVR<br>RVMISRMAKPEEVLIVEDENGEIVRETTKDTDAIALYKTMRETLVFLTHLDTANTEDIMLSKLTLQIDNTEWS<br>WNNLNTLCWAIGSISGAMGEEEERKFLVTVIKELLHLCEIKRGKDNKAVVASNIMYVVGQYPRFLRAHWKFLK<br>TVVNKLFEFMHETHPGVQDMACDTFLKIAQKCRSKFVTLQSGESRPFIVEMLETLPEIIQKLETHQIQSFYES<br>CGCIIASEHDTTTQNQLIMKLFQLPNSSWQQLLYSASFSEDVLRQRDKMKNFSSILRTNSRVALSLGSPYLVQ<br>LEWIYADMLKVYKAYSSMIQALVAGGSQYATKTADARNMRAVKREVLRVIETCISTSQDKDRDQIRKGIVEPL<br>TEPILGDYYSSVADAREPAVLSLFSTVVTYMKGSLSTAEITVIFKSVVGVTLDMIKNNFEDFPDARINFFHLL<br>RSINQHNFSSLFALDDNPAKAEAEFRIIINAIVWAFKHTERNVAETGLQILVEMLRNVDNSPFVNYFYRIYFK<br>SILNDILSVLTDTFHRPGFKLHAQILMHLIGAVASGQITEPIWDQSQPDQVALATGNGSSPPSNAVYLQNHLL<br>KILKDAFPNLTEMQVSDIVKKILSASDEKTFKGHLRDFLVQTKEFSSGDNTDLFDEEKQMLLLEKQKAETERL<br>ARTPGLVAPSNF
Asparagopsis taxiformis Ran-GTP (ATA01349) (176 amino acid residues)
MGVEVHPLKFFTNFGPILMNVWDTAGQEKLGGLRDGYYIQGNAAIIMFDVTSRMTYKNVPQWHRDLVRVCENI<br>PIVLCGNKVDIKDRKVKAKAITFHRKKSLQYYELSAKSNYNFEKPFLWIARKLIGNATLTFVESPALVPPEIK<br>LDNEQIRQIENDINEVRRVPLPDEDDGLDI

Asparagopsis taxiformis AGO3 (ATA08178) (939 amino acid residues)
MPPPKSSSKDSKSKSRPSPSKPSSSSKKPSSSSTPSRHKMDRSSSQAAGPSAMAVDAMRPMSRPGYGRAGKPI<br>KLETNFFPMQIPRSLTVSQYAISIWRYKHPEDVGPQARKSRPRSRPSSSTARSDKKLFEVSASEPIFFNRAVF<br>ALVCRQHGSSFGCSLAYDGRSIAYAARELNRSCLGTMYSVTVDREGHPPTSQDLADGKVEEVKVKIEHAKYLK<br>FDDILQSRVGTIASAEYLAALDVVIATTPLGKYVQIGRSFYSPDNCRPLGKRDSIASAWRGFYQSARLSQLGL<br>VVNLDESFTAFWDGGGRPLADLVQKANGGRELRCDDSRGLREMSTKLKALKVRAQHTRITYRVHGFSSKGANH<br>IMFDSATHGRRVSITEYFMDTYNIRLRHGNQPCVKTNPKRDTYLPMEVLVVMESQRLTGTLSQDQTSSIVKVA<br>SGKPDSRRAAALRTMQRLDHSHDSVCKDFGVKVSSNLITVDARILPTPGIEYSDRRVIKPSQGAWRVNRETFV<br>TGSPLASWAVINACPRLPLEKVQSFVTAVARSAQKNGMTVYNTQPHIYTCRPDRISDEMRSVAARFSKDKRLQ<br>VRQFPLQLIMIIKEKQDTPMYNAIKRTGDLELGIASQCCLEKHVGNDRGGRSREMYCDNLILKINSKLGGQNA<br>AVRGYGADPSSRIPDVPFVNVPHIVLGADVTHPMVGGKSPSVAALVGSRDRQGIQYTGAIRNQPGRQEVIGEI<br>GEMFKEVYSRWRANFGNKHHAASIIMFRDGVSDGQFEEVMQIELEALRRSCRNYNPPFNPRITYIIVTKRHHA<br>RFFGDKNNIDRSGNILPGTVIDQGITSREFYDFYLNSHAGIQGTSKPSKYTVLVDENKIPVDALQGYIFRLAH<br>GFVRCNRSVSMVNSAYYAHLLAFR
Asparagopsis taxiformis HESO1 (ATA06450) (671 amino acid residues)
MWDDPRILVIYRLWTHHISNFMAQPVQFFHIFVMDPQFRQSVFDAGLTFSPETDPSNPVLINLNSTRFGMSER<br>ISALAPPTNPDIRPPNQPPHLPIPTADSPTIHDPRQILSPQQQSPQMLFLSPAVCDLFEAARAATNASAPPTL<br>PIEPPFELIEPAHMLSTSQDSIERLSQHFAETAAVSSPQPENDSARDSAKPHSKSSKKGRPGSNRHPNPQPAE<br>RRLAGRLKVVSRMRNVRIPTTPVCKKQNQTARTEMTQQIDQVFPSISPPDDIASSRADLLARLRKIISDEWPG<br>AGIDIYGSAGNGLGLRSADIDMSLYMPDSVTRSSNGADISAQPRDILARLATIMEENNIAILSKLLEARVPVI<br>KMHDPDSKVQIDVCMNNILAVRNTKLLKAYVDLDPRFRSVCILVKLWAKRRDLNDAYRGTLSSYAYTLLVIHY<br>LQTLDPPVLPCLQATVNGKSVIVGEEQPKEMTDVGHNKLYNTYFDRSVTAATFKSANTGSVCELLLGFFKYFA<br>YTFQYRTDLVSIRLGVRTTREERGWDEQSVYREWEAKQVQFKAAYDVAKAALEARKKADSEERIRLAKLKKEK<br>ANGEASEASSAAGDSTAKRERIVFPRRPRLESKHLFCIEDPFDIDHDLSRGMEKGAVTVIRQEMARAYEILAE<br>SGDFESACEEWKDS
